# Supplementary material for: Frequent Occurrence of NRAS and BRAF Mutations in Human Acral Naevi
Source: Cancers (Basel). 2019 Apr 16;11(4):546. doi: 10.3390/cancers11040546 (PMC6520878; doi:10.3390/cancers11040546)
Supplement: Supplementary file 1 [file cancers-11-00546-s001.pdf]

Supplementary Materials: Frequent Occurrence of *NRAS* and *BRAF* Mutations in Human Acral Naevi

Philipp Jansen, Ioana Cosgarea, Rajmohan Murali, Inga Möller, Antje Sucker, Cindy Franklin, Annette Paschen, Anne Zaremba, Titus J. Brinker, Ingo Stoffels, Dirk Schadendorf, Joachim Klode, Eva Hadaschik and Klaus G. Griewank

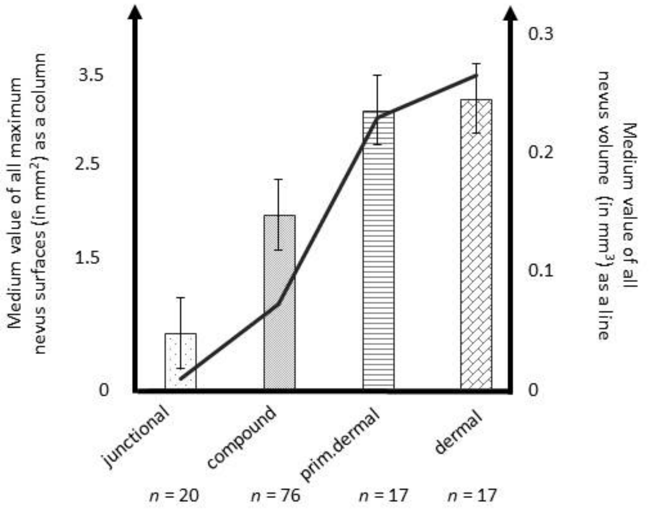

Figure S1. Associations of histological type with size and volume of naevi. Medium size of naevus surface (in mm^2) depicted as columns for junctional, compound, primarily dermal and dermal naevi with standard deviation. Medium volume of naevi for all four locations (junctional, compound, primarily dermal and dermal) is depicted as a black line.

Table S1. Associations of histopathological type of naevus with size (in mm^2).

Size (in mm^2)	Junctional n = 20	Compound n = 76	Primarily Dermal n = 17	Dermal n = 17	p Value *
0–0.5	4	2	3	1	
0.51–1	4	14	1	2	
1.01–1.5	4	8	1	1	
1.51–2	2	17	1	0	
2.01–2.5	0	7	1	2	0.09
2.51–3	2	7	5	4	
3.01–3.5	1	8	1	2	
3.51–4	0	5	2	1	
>4	3	8	2	4	

*chi-square test.

Table S2. Genes covered in the applied sequencing panel.

No.	Gene	Chr.	Location GRCh37	Target Exons	Selection of Relevant Mutations Covered	Primer Pairs
1	*TERT* Prom.	5	1,295,220		228, 242, 250 C>T, CC>TT	1
2	*BRAF*	7	140,453,065	11, 15	G463, G465, V600	4
3	*NRAS*	1	115,256,411	1, 2	G12, G13, Q61	5
4	*HRAS*	11	533,850	1, 2	G12, G13, Q61	2
5	*KRAS*	12	25,380,250	1, 2	G12, G13, Q61	2
6	*KIT*	4	455,593,572	11, 13, 17	L576, K642, N822	3
7	*GNAQ*	9	80,409,369	4, 5	R183, Q209	6
8	*GNA11*	19	3,114,932	4, 5	R183, Q209	3
9	*CYSLTR2*	13	49,281,314	1	L129	1
10	*PLCB4*	20	9,389,740	20	D630	1
11	*SF3B1*	2	198,267,458	14	R625	1
12	*EIF1AX*	X	20,156,647	1, 2	Mutations in exons 1 and 2	3
13	*BAP1*	3	5,243,501	all (17)	Mutations in all exons	46
14	*SRSF2*	17	74,732,226	all (2)	Mutations in all exons	9
15	*GNA14*	9	80,043,813	4, 5	R179, Q205	3
16	*GNA15*	19	3,151,695	4, 5	R186, Q212	4

Table S3. Genes covered in the additional 29 gene sequencing panel (entire gene-all exons—were sequenced).

No.	Gene	Primary Melanoma Type	Customary Mutation Type	Target Bases	Bases Covered	Primer Pairs
1	*BRAF*	cutaneous	activating	2860	2456	40
2	*NRAS*	cutaneous	activating	650	650	10
3	*KIT*	cutaneous	activating	3354	3264	51
4	*HRAS*	cutaneous	activating	780	667	11
5	*KRAS*	cutaneous	activating	787	787	13
6	*CDKN2A*	cutaneous	tumorsuppressor	1184	713	14
7	*PTEN*	cutaneous	tumorsuppressor	1392	1248	22
8	*CDK4*	cutaneous		1052	1052	19
9	*TP53*	cutaneous	tumorsuppressor	1503	1396	26
10	*RAC1*	cutaneous		776	721	14
11	*NF1*	cutaneous	tumorsuppressor	9900	9167	143
12	*PIK3CA*	cutaneous		3607	3313	50
13	*MAP2K2*	cutaneous		1423	1240	24
14	*PIK3R1*	cutaneous		2637	2627	42
15	*MITF*	cutaneous		2066	2066	35
16	*TERT*	cutaneous		3719	2371	39
17	*ARID2*	cutaneous	tumorsuppressor	5928	5830	82
18	*ARID1A*	cutaneous	tumorsuppressor	7258	6132	81
19	*SMARCA4*	cutaneous	tumorsuppressor	5761	5040	88
20	*MAP2K1*	cutaneous		1436	1436	26
21	*CTNNB1*	cutaneous		2626	2626	40
22	*EZH2*	cutaneous		2680	2680	46
23	*IDH1*	cutaneous		1405	1394	22
24	*FBXW7*	cutaneous		2898	2808	43
25	*WT1*	cutaneous		1784	1282	24
26	*GNAQ*	uveal	activating	1220	1064	17
27	*GNA11*	uveal	activating	1220	944	14
28	*BAP1*	uveal	tumorsuppressor	2599	2380	39
29	*SF3B1*	uveal		4455	4412	72

© 2019 by the authors. Licensee MDPI, Basel, Switzerland. This article is an open access article distributed under the terms and conditions of the Creative Commons Attribution (CC BY) license (http://creativecommons.org/licenses/by/4.0/).
